# Kruppel-Like Factor 15 Is Critical for the Development of Left Ventricular Hypertrophy

**DOI:** 10.3390/ijms19051303

**Published:** 2018-04-27

**Authors:** Sheila K. Patel, Jay Ramchand, Vincenzo Crocitti, Louise M. Burrell

**Affiliations:** 1 Department of Medicine, Austin Health, University of Melbourne, Melbourne, VIC 3084, Australia; jay.ramchand@unimelb.edu.au (J.R.); v.crocitti@student.unimelb.edu.au (V.C.); l.burrell@unimelb.edu.au (L.M.B.); 2 Department of Cardiology, Austin Health, Melbourne, VIC 3084, Australia

**Keywords:** Kruppel-like factor 15, left ventricular hypertrophy, cardiac hypertrophy, heart failure, genetics of left ventricular hypertrophy

## Abstract

Left ventricular hypertrophy (LVH) is an independent risk factor for adverse cardiovascular events and is often present in patients with hypertension. Treatment to reduce blood pressure and regress LVH is key to improving health outcomes, but currently available drugs have only modest cardioprotective effects. Improved understanding of the molecular mechanisms involved in the development of LVH may lead to new therapeutic targets in the future. There is now compelling evidence that the transcription factor Kruppel-like factor 15 (KLF15) is an important negative regulator of cardiac hypertrophy in both experimental models and in man. Studies have reported that loss or suppression of KLF15 contributes to LVH, through lack of inhibition of pro-hypertrophic transcription factors and stimulation of trophic and fibrotic signaling pathways. This review provides a summary of the experimental and human studies that have investigated the role of KLF15 in the development of cardiac hypertrophy. It also discusses our recent paper that described the contribution of genetic variants in *KLF15* to the development of LVH and heart failure in high-risk patients.

## 1. Introduction

Left ventricular hypertrophy (LVH) is an independent and potent risk factor for cardiovascular events [1] and is often present in patients with hypertension. LVH is diagnosed by electrocardiography, echocardiography or cardiac magnetic resonance imaging. The prevalence of LVH in the general population is 10% [1] but rises to 40–70% in those with risk factors such as hypertension, aortic stenosis, diabetes and chronic kidney disease [2,3,4,5]. As LVH per se causes no symptoms (preclinical), it is often only diagnosed after patients present with symptoms such as heart failure [1]. Treatment to regress LVH is key to improving health outcomes [6,7], but currently available drugs have only modest cardioprotective effects [8]. Improved understanding of the molecular mechanisms that contribute to the development of LVH provides an opportunity to identify pathways that may be new therapeutic targets.

**Figure 1:**
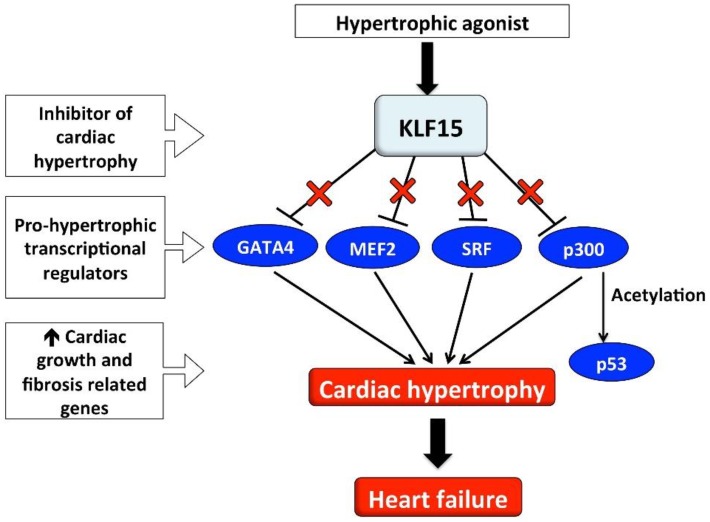
Schema illustrating how loss of the inhibitory effect of KLF15 on pro-hypertrophic cardiac transcriptional regulators may contribute to the development of LVH and heart failure. **↑** Increased; X in red font represents the inhibitory effect; GATA4, GATA binding protein 4; KLF15, Kruppel-like factor 15; MEF2, myocyte enhancer factor 2; SRF, serum response factor.

Our group is interested in the function and regulation of transcription factors in the development of LVH. To date, studies have mainly focused on factors that induce hypertrophy such as GATA binding protein 4 (GATA4), myocyte enhancer factor 2 (MEF2) and serum response factor (SRF) [9] (Figure 1). However, there are also transcriptional repressors of hypertrophy such as Kruppel-like factor 15 (KLF15) [10], and compelling experimental evidence that suppression of KLF15 contributes to LVH through lack of inhibition of pro-hypertrophic transcription factors [9]. This review provides a summary of experimental and human studies that have investigated the role of KLF15 in the development of cardiac hypertrophy. It also discusses our recent paper that described the contribution of genetic variants in *KLF15* to the development of LVH and heart failure in high-risk patients.

## 2. Kruppel-Like Factor 15 and Cardiac Hypertrophy

KLF15 is one member of a family of 18 Kruppel-like factors (KLF). The KLFs are a subclass of the zinc-finger family of DNA-binding transcriptional regulators, homologous to the *Drosophila* gap gene Kruppel [11]. KLF15 is expressed in myocytes and fibroblasts [12]. Both in vitro and in vivo studies support a role for KLF15 as a repressor of pathological cardiac hypertrophy and fibrosis [10,12,13]. Cardiac KLF15 expression is low during development and increases after birth to reach high levels in the adult rat and mouse heart [13,14]. The action of KLF15 to repress hypertrophy occurs through the inhibition of the activity of pro-hypertrophic transcriptional regulators, which affect atrial natriuretic peptide (ANP) and brain natriuretic peptide (BNP) promoter activity [13]. Expression of KLF15 is unchanged during physiologically induced LVH (i.e., exercise) but is decreased in pathological LVH [15].

Table 1 summarizes the results from in vivo studies that have explored KLF15 in experimental models of cardiac hypertrophy. Overall, regardless of the model used, the results have consistently shown that loss of KLF15 contributes to the development of cardiac hypertrophy. The approaches used to induce LVH include cardiac KLF15 null (−/−) mice, high blood pressure (angiotensin (Ang) II infusion [10], transgenic TGR(mRen2)27 rat (Ren-2) [15], high salt diet in Dahl salt-sensitive rats, isoproterenol infusion [16]) and surgical methods such as transaortic constriction [13], ascending aortic constriction [12,13], and aortic banding [17].

### 2.1. Mouse Models of KLF15 Gene Deletion, Overexpression and Cardiac Hypertrophy

KLF15 null mice are viable with increased heart cavity size and reduced left ventricular (LV) fractional shortening, but no increase in wall thickness [13]. Under conditions of pressure–overload due to ascending aortic constriction, KLF15 (−/−) null mice had marked LV cavity dilation, reduced systolic function, and increased cardiac mass compared to KLF15 wildtype (+/+) mice. Morphometric studies indicated that cardiomyocytes from KLF15 null mice were larger and longer compared to cardiomyocytes from wildtype mice with ascending aortic constriction. Others have reported that ascending aortic constriction in KLF15 null mice led to increased profibrotic factor connective tissue growth factor (CTGF) expression compared to sham operated KLF15 null mice [12]. Similar effects were seen in the study by Halder et al. [10] in which Ang II infusion led to impaired LV dysfunction and cavity dilation with increased cardiac mass in KLF15 null mice; the observed effects were not related to differences in blood pressure between wild type and null mice. Isoproterenol infusion also increased cardiac mass, cardiomyocyte cross-sectional area and led to significant fibrosis in KLF15 null mice compared to vehicle treated KLF15 null mice [16], with no effect in wildtype KLF15 mice [16]. Pressure-overload in mice with genetic deletion of KLF15 also led to a parallel increase in the cardiac expression of hypertrophic markers ANP, BNP and CTGF [10,12,13,16].

Halder et al. [10] examined the effect of restoring KLF15 levels on cardiac hypertrophy. The authors hypothesized that p53, a protein known to regulate the expression of genes involved in growth and apoptosis and activated by Ang II, played a role in cardiac decompensation. They reported that KLF15 null mice had a significant increase in p53 expression after Ang II infusion, and that KLF15 deficient hearts were rescued by p53 deletion or with curcumin, a potent p300 acetyltransferase inhibitor [10]. The p300 acetyltransferase inhibitor is an important regulator of p53 function and involved in acetylating GATA4, MEF2 and the Smads. Curcumin also ameliorated heart failure in KLF15 null mice, reduced cardiac mass, improved LV systolic function and decreased p53 abundance in heart tissue. The authors also investigated the effects of adenoviral KLF15 overexpression in neonatal rat ventricular cardiomyocytes (NRVM), which did not reduce p300 abundance or acetyltransferase activity [10].

The therapeutic potential of KLF15 overexpression on LVH in mice was examined using recombinant adenovirus (AAV9) to overexpress KLF15 and Ang II infusion to stimulate cardiac hypertrophy [18]. As expected Ang II infusion increased LV mass and cardiomyocyte size in AAV- green fluorescent protein (GFP, control vector) mice, with a blunted increase in LV mass and no change in cardiomyocyte size in Ang II infused mice infected with AAV9-KLF15. Ang II infusion increased interstitial fibrosis and LV mRNA expression of hypertrophic marker genes ANP and alpha skeletal actin (αSKA) in AAV9-GFP mice, but this effect was not modulated in the AAV9-KLF15 mice despite the reduction in LV mass. This result suggests that collagen deposition may occur through pathways independent of KLF15 and its effect to inhibit cardiac hypertrophy.

### 2.2. Rat Models of Cardiac Hypertrophy and KLF15

KLF15 expression was examined in 2 models of hypertension—the transgenic TGR(mRen2)27 rat (Ren-2) [15] and the high salt fed Dahl salt-sensitive rat [19]. The Ren-2 rat is characterized by severe hypertension, LVH, suppression of the kidney renin-angiotensin system and unchanged or suppressed levels of plasma renin, Ang I and II and angiotensinogen compared to wildtype rats [20]. Cardiac KLF15 expression was reduced at an early stage of LVH. In an elegant series of experiments, cardiac biopsies were taken from the rats with a similar degree of cardiac hypertrophy and normal systolic function, and followed over time. KLF15 mRNA expression decreased more in the hypertrophied hearts that progressed to heart failure compared to control and compensated rats, suggesting that expression of KLF15 may play a role in the prevention of heart failure.

Dahl salt-sensitive rats fed a high salt diet developed hypertension-induced LVH at 11 weeks, with LV dilatation and heart failure at 17 weeks [19]. Cardiac KLF15 mRNA was significantly reduced with LVH and decreased further with the development of heart failure. This change was associated with increased ANP gene expression with LVH and a further increase in levels with the development of heart failure [19].

Aortic stenosis is a common cause of LVH and KLF15 expression has been examined in two experimental models of aortic constriction—transaortic constriction and aortic banding. In adult Sprague-Dawley rats, KLF15 mRNA expression was non-significantly reduced at 2 days post transaortic constriction with a significant reduction by 7 days post transaortic constriction [13].

Yu et al. [17] conducted studies in rats with pressure overload secondary to aortic banding and examined the effect of debanding and unloading pressure at 3 and 6-weeks. Aortic banding increased ejection fraction and LV pressure compared to the sham group, and both improved after debanding. At both 3 and 6-weeks after aortic banding, rats had decreased KLF15 mRNA, and increased CTGF, transforming growth factor-β (TGFβ) and myocardin-related transcription factor A (MRTF-A) with fibrosis and increased collagen I and III mRNA compared to sham-operated rats. KLF15 levels dropped further in the 6-week banded animals. Aortic debanding resulted in a decrease in collagen mRNA, increased KLF15 and reduced TGFβ mRNA.

### 2.3. In Vitro KLF15 and LVH Studies

Table 2 summarizes the results of *in vitro* studies of KLF15 which have been mainly conducted in NRVM, and include *KLF15* gene silencing [15], KLF15 overexpression [13,15], and cardiomyocyte hypertrophy induced with pro-hypertrophic factors [13,15]. Two studies were conducted in cardiac fibroblasts [12,17].

### 2.4. KLF15 Knock Down or Overexpression

In rat cardiomyocytes treated with KLF15 siRNAs, there were significant increases in cardiomyocyte size and ANP mRNA expression [15]. The loss of KLF15 alone was sufficient to induce cell hypertrophy [13]. Two studies explored overexpression of KLF15 in cardiomyocytes [13,15]. In contrast to the gene knock down study, overexpression of KLF15 reduced cardiomyocyte ANP and BNP mRNA [13,15] and led to reduced cardiomyocyte size in one study [13] but not the other [15].

Two studies examined KLF15 levels in cardiac fibroblasts. KLF15 inhibited the expression of CTGF in cardiac fibroblasts [12,17]. Adenoviral overexpression of KLF15 in neonatal rat ventricular fibroblasts (NRVF) inhibited both basal and TGFβ induced CTGF expression. Similar to TGFβ treatment in cardiomyocytes [15], TGFβ reduced KLF15 gene expression and induced CTGF in NRVF [12]. Co-immunoprecipitation and mobility shift experiments showed KLF15 inhibited recruitment of a co-activator P/CAF (a potent transcriptional co-activator of Smad3 target genes) to the CTGF promoter with no effect on Smad3 binding. KLF15 mediated repression of CTGF promoter was rescued by overexpressing P/CAF [12].

In primary neonate rat cardiac fibroblasts in which hypertrophy was also induced with TGFβ, silencing of the KLF15 gene with KLF15-shRNA significantly decreased KLF15 protein. In contrast, KLF15 overexpression with a recombinant adenovirus increased KLF15 protein expression. Fibroblasts stimulated with TGFβ alone showed increased collagen mRNA. TGFβ stimulated fibroblasts with KLF15 overexpression had less fibrosis and hypertrophy, reduced CTGF and myocardin related transcription factor A (MRFT-A) mRNA, all of which were increased in KLF15-shRNA infected fibroblasts [17].

### 2.5. Hypertrophy Induced by Stimulation with Pro-Hypertrophic Factors

KLF15 mRNA expression is low in NRVM but induced with serum starvation with an expression pattern that is antiparallel to that of ANP and BNP gene expression [13]. Treatment of NRVM with the pro-hypertrophic stimuli, endothelin-1 and phenylephrine, decreased KLF15 mRNA expression and induced ANP and BNP expression. Similarly, in cultured neonatal rat cardiomyocytes, stimulation with phenylephrine, endothelin-1 and TGFβ significantly reduced KLF15 expression levels [15]. However KLF15 expression was unchanged when cardiomyocyte growth was induced with physiological hypertrophy stimuli (i.e., insulin, insulin-like growth factor 1 or 2). These results support a role for the involvement of KLF15 in pathological hypertrophy but not in physiological hypertrophy. TGFβ-mediated down regulation of KLF15 was abolished with knockdown of the TGFβ receptor 1 [15]. TGFβ-mediated activation of p38 mitogen-activated protein kinase was also necessary and sufficient to decrease KLF15 expression. Adenoviral overexpression of an upstream p38 kinase (MKK6) that induced increased p38 phosphorylation led to an 80% decrease in KLF15 mRNA and the induction of BNP. No other studies have explored TGFβ stimulated hypertrophy and KLF15 levels in cardiomyocytes.

## 3. Human Studies of KLF15

### 3.1. KLF15 Expression in Human Cardiac Tissue

Consistent with the observation in experimental studies, there is evidence that loss of cardiac KLF15 expression may contribute to pathological LVH in humans (Table 3). Three separate studies investigated KLF15 expression in LV tissue obtained from patients undergoing cardiac surgery. In patients with LVH secondary to aortic stenosis, KLF15 protein was reduced in myocardial needle biopsy samples taken from the anterior LV of patients undergoing open heart surgery (*n* = 8) compared to patients undergoing coronary bypass grafting (*n* = 6) [13]. These patients were selected from a larger group of patients from an earlier study [21]. The characteristics of the subgroup of patients used in the paper by Fisch et al. [13] were not provided. KLF15 mRNA was also significantly reduced in LV samples of patients with non-ischaemic cardiomyopathy (*n* = 36) compared to control tissue from non-failing hearts deemed unsuitable for transplantation (*n* = 30) [10]. 

In patients with end-stage heart failure undergoing implantation and explantation of a left ventricular assist device (LVAD) as a bridge to transplantation, pre-device implantation LV KLF15 mRNA expression was reduced in the failing heart compared to control non-failing hearts, with significant recovery of KLF15 expression after mechanical unloading [14]. The latter study used 3–4 samples per group, collected as part of a larger study (36 pre-LVAD, 30 post-LVAD) [22].

### 3.2. Genetic Studies of KLF15 in Patients with LVH

Conventional risk factors do not explain all the variability in LVH [23], and a heritable component underlying LVH is recognized. We recently explored the genetic association of *KLF15* single nucleotide polymorphisms (SNP) in patients with type 2 diabetes and an echocardiographic assessment of LV mass [2]. The intronic *KLF15* SNP rs9838915 A allele was significantly associated with LV mass and the association was replicated in an independent cohort of >5000 type 2 diabetes patients (Table 3). The *KLF15* rs9838915 A allele predicted the first hospitalization with heart failure [24]. No other genetic studies have been conducted in this area. Analysis of the GTEx data, [25], identified 84 expression quantitative trait loci downstream of *KLF15* as highly significant (*p* values 0.00011 to 3.8^10−8^, false discovery rate <5%, alternative allele effect sizes from 0.12–0.16) modifiers of KLF15 gene expression in transformed fibroblasts. Although the rs9838915 SNP was not examined in GTEx, the data suggest that *KLF15* SNPs have the functional potential to influence KLF15 expression. Recently, Ferreira et al. [26] conducted a bioinformatics analysis focused on non-synonymous variants in KLF genes using algorithms to predict the effect of variants on the genes structure and function. Two *KLF15* intronic variants, one located in the conserved Zinc-finger domain and the other in a non-conserved region was predicted to affect DNA binding or protein destabilization and hence may lead to disrupted KLF15 protein function. The frequency of these variants is <0.0001 in the population and therefore unlikely to contribute to LVH in the general population.

## 4. Proposed Mechanisms: KLF15 and Cardiac Hypertrophy

A number of mechanisms by which KLF15 contributes to cardiac hypertrophy have been proposed. Gene reporter studies show that KLF15 inhibits MEF2 and GATA4 DNA-binding transcriptional activation and that KLF15 negatively regulates cardiac hypertrophy by preventing DNA-binding of MEF2 and GATA4 to their transcriptional targets [13]. KLF15 activation represses the acetyltransferase p300 mediated acetylation of p53 to maintain normal heart function [10]. Conversely, KLF15 deficiency leads to hyperacetylation of p53 in the mice hearts and in human hearts. This action of KLF15 is important as p300 acetylates several pro-hypertrophic markers involved in pathogenic remodeling including GATA4, MEF2 and Smad. Furthermore, p300 is a direct transcriptional target of TGFβ in fibroblasts and its levels are elevated in experimental models of fibrosis [27]. The KLF15 protein domain map shows highly conserved regions between residues 140–160 in the putative p300-interacting transactivation domain and conserved sequences in this region between species [14]. In the same study, KLF15 deficient mice were rescued by p53 deletion or p300 inhibition by curcumin. The authors propose that loss of KLF15 is not a response to heart failure but that loss of KLF15 contributes to progression to heart failure by removing the ability to repress key cardiac transcription factors that enable growth [10].

KLF15 competitively inhibits SRF binding to the transcriptional co-activator myocardin thus preventing expression of cardiotrophic genes such as ANP [15]. GST-pulldown assays revealed no direct interaction between KLF15 and SRF or MEF2 [15] suggesting previous interactions reported may be indirect [13]. KLF15 also competitively inhibits binding of MRTF-A and MRTF-B to SRF, repressing SRF-dependent cardiotrophic gene expression [18]. CTGF, a key mediator of fibrosis in pathological cardiac hypertrophy [28], is negatively regulated by KLF15 and increased TGFβ levels activate p38-MAPK signaling which downregulates KLF15 leading myocardin to bind to SRF and to activation of cardiotrophic genes [15]. Therefore, KLF15 acts as a negative repressor of cardiac hypertrophy via inhibitory competitive binding to myocardin [15,18].

Tandler et al. [29] postulated that KLF15 deficiency could impair the mitochondrial division process, creating enlarged mitochondria that contribute to hypertrophic cardiomyocytes. KLF15 deficient mice had sparse dispersions of megamitochondria within LV tissue on electron microscopy [29]. Although normal morphological mitochondria were more frequently observed, megamitochondria were up to three times wider and significantly longer in length with morphological features suggestive of inability to undergo fission. Others reported no differences in mitochondrial ultrastructure in wild-type and knock-out KLF15 mice on electron microscopy [14]. A role for KLF15 in cardiac metabolism has also been suggested. KLF15 positively influences the energetics of cardiac metabolism and KLF15 null mice have impaired mitochondrial fatty acid [14].

## 5. Summary

In the past decade, there has been increasing evidence from both experimental and human studies that KLF15 is an important regulator of cardiac hypertrophy. The studies clearly support a pathological role of KLF15 in the development of cardiac hypertrophy and identify KLF15 as part of a transcriptional pathway regulating the cardiac response to hypertrophic stimuli. Thus, loss of KLF15 leads to transcription repression and stimulates fibrotic signaling pathways. Overexpression studies suggest KLF15 inhibits hypertrophy and reduces cardiomyocyte size and pro-hypertrophic and pro-fibrotic markers such as ANP, BNP and CTGF. Genetic variants in *KLF15* may also contribute to the development of LVH and predict heart failure outcomes in those with LVH. The genetic findings complement the human studies which reported reduced KLF15 expression in the hearts of patients with LVH and heart failure. The only suggested therapeutic pathway for preventing loss of KLF15 and transition to heart failure thus far has been inhibition of the p53 and p300 pathway.

## Figures and Tables

**Table 1:** In vivo KLF15 experimental studies.

Experiment	Model/Animal Strain	Control Group	Summary of Main Findings	References
Genetic models	Ascending aortic constriction in KLF15 (−/−) and KLF15 (+/+) C57BL/6 mice	Sham-operation	KLF15 (−/−) vs. (+/+) - ↓ LV FS, ↑ cavity size; AAC in KLF15 (−/−) led to ↑ LV cavity dilation, ↓ systolic function,↑ cardiac mass, ↑ cardiomyocyte cell size, ↑ ANF and BNP mRNA	Fisch S et al., 2007 [13]
	Ascending aortic constriction in KLF15 (−/−) and KLF15 (+/+) C57BL/6 mice	Sham-operation	AAC led to ↑ cardiac CTGF mRNA in KLF15 (−/−); no change in controls	Wang B et al., 2008 [12]
	Ang II infusion in KLF15 (−/−) C57BL/6 mice	Saline infusion in KLF15 (+/+) mice	Ang II led to ↑ cardiac mass and cavity dilation, ↓ systolic function, ↓ LV KLF15 mRNA, ↑ ANF expression vs. control	Halder et al., 2010 [10]
Hypertension induced LVH	High salt diet in Dahl salt-sensitive rats; LVH at 11 weeks and heart failure at 17 weeks	Age-matched low salt diet	Further ↓ cardiac KLF15 mRNA as LVH progressed to heart failure; ↑ ANP with LVH and further ↓ with heart failure	Horie T et al., 2009 [19]
	Time course of cardiac biopsies in hypertensive transgenic TGR(mRen2)27 rats (Ren-2)	Wildtype littermates	Cardiac KLF15 mRNA ↓ with progression from LVH to heart failure	Leenders et al., 2010 [15]
	14-day Ang II infusion in C57BL/6 male mice.	Saline infusion	↓ KLF15 mRNA expression in ventricle	Halder et al., 2010 [10]
	Adenoviral (AAV9) KLF15 or GFP (control vector) over-expression in 8-week-old C57BL/6 mice + 28-day Ang II infusion	Saline infusion	↑ Interstitial fibrosis in both groups compared to controls; ↓ cardiac hypertrophy and cardiomyocyte area in AAV9-KLF15/Ang II vs. AAV9-GFP/Ang II	Leenders et al., 2012 [18]
	5-week isoproterenol or vehicle infusion in KLF15 (−/−) C57BL/6 mice	KLF15 (+/+) mice	↑ Cardiac mass, ↑ cardiomyocyte cross-sectional area, ↑ fibrosis in KLF15 (−/−) isoproterenol vs. vehicle; ↔ cardiac mass, cell size and fibrosis in KLF15 (+/+) isoproterenol vs. vehicle	Gao et al., 2017 [16]
Surgical induction of LVH	Pressure-overload hypertrophy induced by TAC in adult male Sprague-Dawley rats	No control group	↓ KLF15 LV mRNA expression at 2-days post-TAC and further ↓ at 7-days post-TAC	Fisch S et al., 2007 [13]
	Aortic banding in Sprague-Dawley rats with debanding at 3 and 6-weeks post-surgery	Time-matched sham-operated rats	↓ Cardiac KLF15 mRNA, ↑ interstitial fibrosis, ↑ CTGF and ↑ TGFβ mRNA at 3- and 6-week post-banding; debanding led to ↑ cardiac KLF15 and ↓ TGFβ mRNA	Yu et al., 2014 [17]

**↑** Increased; **↓** decreased; **↔** no change. AAC, ascending aortic constriction; AAV9, adeno-associated virus 9; Ang II, angiotensin II; ANP, atrial natriuretic peptide; BNP, brain natriuretic peptide; CTGF connective tissue growth factor; FS, fractional shortening; GFP, green fluorescent protein; KLF15, Kruppel-like factor 15; LV, left ventricle; LVH, left ventricular hypertrophy; mRNA, messenger ribonucleic acid; TAC, transaortic constriction; TGFβ, transforming growth factor beta; TGR(mREN2)27, transgenic renin hypertensive model.

**Table 2:** In vitro KLF15 studies.

Experiment Type	Experimental Model/Cell	Control Group	Summary of Main Findings	References
KLF15 gene silencing	Neonatal rat (Lewis) and mouse (FBV mice) cardiomyocytes treated with 2 siRNAs against KLF15	Cells with non-targeted control siRNA	↑ Cardiomyocyte size, ↑ANP mRNA	Leender et al., 2010 [15]
	Neonatal Sprague-Dawley rat primary cardiac fibroblasts with viral KLF15 gene silencing. Hypertrophy induced with TGFβ	Fibroblasts either without TGFβ stimulation or control virus	↓ KLF15 mRNA	Yu et al., 2014 [17]
KLF15 overexpression	Adenoviral overexpression of KLF15 in NRVM	GFP control vector	↓ ANP and BNP mRNA; ↓ cardiomyocyte size under basal and phenylephrine stimulated hypertrophy	Fisch S et al., 2007 [13]
	Lentiviral overexpression of KLF15 in neonatal rat (Lewis) cardiomyocytes	Cells with control vector	↓ ANP mRNA, ↔ cardiomyocyte size	Leender et al., 2010 [15]
	Neonatal Sprague-Dawley rat primary cardiac fibroblasts with adenoviral KLF15 overexpression. Hypertrophy induced with TGFβ.	Fibroblasts either without TGFβ stimulation or control virus	↑ KLF15 protein, ↓ fibrosis and hypertrophy, ↓ CTGF mRNA with TGFβ stimulation and KLF15 overexpression	Yu et al., 2014 [17]
	Adenoviral overexpression of KLF15 in NRVF	GFP control vector	Inhibits basal and TGFβ induced CTGF expression	Wang et al., 2008 [12]
Cardiac hypertrophy induced by pro-hypertrophic stimuli	Isolated NRVM. Stimulation of hypertrophy with phenylephrine, endothelin-1.	No controls	↓ KLF15 and ↑ANP and BNP mRNA expression with pro-hypertrophic stimuli	Fisch S et al., 2007 [13]
	Isolated NRVF 2 day old Sprague-Dawley rats stimulated with TGFβ	NRVF under basal conditions	↓ KLF15 and ↓ CTGF mRNA post TGFβ1 stimulation	Wang et al., 2008 [12]
	Neonatal rat (Lewis) LV cardiomyocytes. Stimulation of hypertrophy (phenylephrine, endothelin-1, TGFβ) or by stimuli known to stimulate physiological hypertrophy (insulin, IGF-1, IGF-2)	Control cells	↓ KLF15 expression with all hypertrophic stimuli. ↔ KLF15 expression with physiological growth. TGFβ knockdown abolished ↓ KLF15	Leender et al., 2010 [15]
	Neonatal rat (Lewis) and mouse (FBV mice) cardiomyocytes, treated with two p38 MAPK inhibitors and TGFβ stimulation	Cells without p38 MAPK inhibitor or TGFβ stimulation.	TGFβ – induced ↓ KLF15 expression abolished by p38 MAPK inhibitors	Leender et al. 2010 [15]

**↑** Increased; **↓** decreased; **↔** no change. Ang II, angiotensin II; ANP, atrial natriuretic peptide; BNP, brain natriuretic peptide; CTGF, connective tissue growth factor; GFP, green fluorescent protein; IGF-1, insulin-like growth factor 1; IGF-2, insulin-like growth factor 2; KLF15, Kruppel-like factor 15; LV, left ventricle; MAPK, mitogen-activated protein kinase; mRNA, messenger ribonucleic acid; NRVF, neonatal rat ventricular fibroblasts; NRVM, neonatal rat ventricular myocytes; TGFβ, transforming growth factor beta.

**Table 3:** Human studies of KLF15.

Population Group and Sample Size (*n*)	Summary of Main Findings	References
LV tissue from patients with LVH due to aortic stenosis (*n* = 8) and control subjects undergoing CABG without LVH (*n* = 6).	KLF15 protein detected in nuclei of myocytes from control patients. ↓ cardiac KLF15 protein in LVH due to aortic stenosis	Fisch S et al. 2007 [13]
LV tissue from heart transplant patients with systolic heart failure (non-ischemic cardiomyopathy, *n* = 36) and non-failing control hearts (*n* = 30).	↓ cardiac KLF15 mRNA in failing hearts	Halder et al. 2010 [10]
LV tissue taken pre- and post-LVAD implantation/explantation in end-stage heart failure patients and non-failing hearts. (*n* = 3–4/group)	↓ cardiac KLF15 mRNA pre-LVAD compared to control non-failing hearts. ↑ cardiac KLF15 mRNA expression post LVAD	Prosdocimo et al. 2014 [14]
Type 2 diabetes patients (*n* = 318) with LVH and heart failure outcomes; replication LVH cohort (*n* = 5631)	↑ LV mass and LVH with *KLF15* SNP rs9838915 A allele; finding replicated in independent cohort. *KLF15* rs9838915 A allele predicted development of first hospitalization with heart failure	Patel et al. 2017 [24]

**↑** Increased; **↓** decreased. CABG, coronary artery bypass graft; mRNA, messenger ribonucleic acid; LV, left ventricle; LVAD, left ventricular assisted device; LVH, left ventricular hypertrophy; KLF15, Kruppel-like factor 15; SNP, single nucleotide polymorphism.
